# One or two things we know about concept drift—a survey on monitoring in evolving environments. Part A: detecting concept drift

**DOI:** 10.3389/frai.2024.1330257

**Published:** 2024-06-19

**Authors:** Fabian Hinder, Valerie Vaquet, Barbara Hammer

**Affiliations:** Faculty of Technology, Bielefeld University, Bielefeld, North Rhine-Westphalia, Germany

**Keywords:** concept drift, drift detection, monitoring, survey, data streams

## Abstract

The world surrounding us is subject to constant change. These changes, frequently described as concept drift, influence many industrial and technical processes. As they can lead to malfunctions and other anomalous behavior, which may be safety-critical in many scenarios, detecting and analyzing concept drift is crucial. In this study, we provide a literature review focusing on concept drift in unsupervised data streams. While many surveys focus on supervised data streams, so far, there is no work reviewing the unsupervised setting. However, this setting is of particular relevance for monitoring and anomaly detection which are directly applicable to many tasks and challenges in engineering. This survey provides a taxonomy of existing work on unsupervised drift detection. In addition to providing a comprehensive literature review, it offers precise mathematical definitions of the considered problems and contains standardized experiments on parametric artificial datasets allowing for a direct comparison of different detection strategies. Thus, the suitability of different schemes can be analyzed systematically, and guidelines for their usage in real-world scenarios can be provided.

## 1 Introduction

The constantly changing world presents challenges for automated systems, for example, those involved in critical infrastructure, manufacturing, and quality control. Reliable functioning of automated processes and monitoring algorithms requires the ability to detect, respond, and adapt to these changes (Ditzler et al., 2015; Reppa et al., 2016; Chen and Boning, 2017; Vrachimis et al., 2022; Gabbar et al., 2023).

Formally, changes in the data-generating distribution are known as *concept drift* (Gama et al., 2014). These changes can be caused by modifications in the observed process, environment, or data-collecting sensors. Detecting anomalies in the observed process is essential for identifying faulty productions or other types of unwanted errors. Conversely, detecting changes in sensors and the environment is crucial for automated processes to take appropriate actions, such as replacing a faulty sensor or modifying the system processing the collected data to fit a new scenario (Gama et al., 2004, 2014; Gonçalves et al., 2014).

Typically, drift is studied in *stream setups*, where changes in the underlying data distribution necessitate model adaptation or alerting a human operator for corrective action (Ditzler et al., 2015; Lu et al., 2018; Delange et al., 2021). This is closely linked to the evolution of concepts in *continual learning*, a widespread subject in deep learning where concepts can arise or vanish. Drift extends beyond data streams and appears in *time-series* data with interdependent observations. Such drift usually manifests itself as trends, and its absence is known as *stationarity* (Esling and Agon, 2012; Aminikhanghahi and Cook, 2017).

In settings where data are observed over time, such as manufacturing and quality control, data are frequently gathered across multiple locations and subjected to *federated learning* techniques (Zhang et al., 2021). Instead of consolidating all data on a global server, local processing is implemented, and outcomes are integrated into an overarching model. Similar to stream learning, it is crucial to address differences or drift in data from various locations to build a strong global model (Liu et al., 2020). Furthermore, drift must be taken into account in *transfer learning*, a deep learning technique (Pan and Yang, 2010) in which the model is pre-trained on a similar task with a more extensive dataset before being fine-tuned on the target task using a limited dataset. Although the main focus of this study is on data streams, the strategies presented herein apply to other tasks.

Processing drifting data streams involves two major tasks: establishing a robust model for predictive tasks, that is, *online or stream learning*, and *monitoring* systems for unexpected behavior. In the former, the focus is on a *label* and its relation to other features, while the latter is concerned with any change indicating unexpected system behaviors or states. Drift detection, therefore, focuses on different goals, in analogy to general learning termed *supervised* for the former and *unsupervised* for the latter. This study omits online learning as it has been extensively explored in previous surveys (Ditzler et al., 2015; Losing et al., 2018; Lu et al., 2018) and toolboxes (Bifet et al., 2010; Montiel et al., 2018, 2021).

Instead, this study centers on unsupervised drift detection and monitoring situations where drift is anticipated due to sensor usage or sensitivity to environmental changes. Specifically, the focus is on unsupervised drift detection, which is vital for monitoring and comprehending drift phenomena. Some exemplary applications are the detection of drift for security applications (Yang et al., 2021) and the usage of drift detection for the detection of leakages in water distribution networks (Vaquet et al., 2024a,b). In addition, there are techniques for further analyzing drift (Webb et al., 2017, 2018; Hinder et al., 2023a), which we will not cover in detail in this study. For the interested reader, we provide an extended version that covers these topics as well as the content of this study (Hinder et al., 2023b). Note that approaches for unsupervised drift detection discussed here differ from those designed for online learning, as discussed by Gemaque et al. (2020). In Section 2.2, we describe the contrast to supervised drift detection in more detail.

Monitoring entails observing a system and offering necessary information to both human operators and automated tasks to ensure proper system functionality. The required information varies depending on the specific task (Goldenberg and Webb, 2019; Verma, 2021). Generally, there are crucial inquiries to answer regarding drift (Lu et al., 2018):

The first one pertains to the *whether (and when)* of drift occurrence, which is addressed through *drift detection* (Gama et al., 2014). When detecting drift, a precise assessment of its severity, that is, the *how much?*, is crucial in determining appropriate measures. *Drift quantification*, estimating the rates of change that trigger alarms, often precedes detection, and although not the main focus, this aspect will be briefly discussed later.

To take accurate action, it is essential to pinpoint drift more precisely (Lu et al., 2018). While detecting and quantifying drift addresses the *when* by identifying change points and rate of change, *drift localization* and *segmentation* (Lu et al., 2018) focus on the *where* by assigning drift-related information to the data space. For example, identifying anomalous items, specifically drifting data samples, is crucial in monitoring settings.

Addressing the aforementioned issues may not be sufficient in some cases. Systems can experience drift, a malfunction resulting in changes across multiple data points and features. For example, a deteriorating sensor can produce altered measurements. Reliance solely on drift location provides limited insight into the nature of the event. However, it is crucial to provide detailed information about what happened and how it occurred. In many cases, *drift explanations* (Hinder et al., 2023a) provide relevant information to human operators concerned with monitoring and manual model adaptation. Finding appropriate explanations is crucial since the complexity of the drift may go beyond the information obtained by answering the previously raised questions.

This study is organized as follows: First, we formalize the concept of drift (Section 2.1) and position our work in the context of related research at the intersection of the stream setup, supervised, and unsupervised approaches (Section 2.2). We then turn our attention to drift detection: We begin by formalizing the task (Section 3) and presenting a general scheme implemented by most approaches (Section 4). We then discuss and categorize several detection methods (Section 5) and perform an analysis based on criteria specific to drift and streaming scenarios (Section 6). In the ArXive version (Hinder et al., 2023b), we also cover topics that are closer related to the analysis of concept drift like drift localization and drift explanation.

## 2 Concept drift—defining the setup

In this section, we first formally define drift. Then, we explore various setups for dealing with drift before delving into a detailed examination of the body of work covering drift detection approaches in the later sections.

### 2.1 A formal description of concept drift

In classical batch machine learning, one assumes that the distribution remains constant during training, testing, and application. We denote this time-invariant data-generating distribution by D and consider a sample of size *n* is a collection of *n* i.i.d. random variables $X_{1},\ldots ,X_{n}\sim D$.

However, real-world applications, particularly stream learning, often violate the assumption of time-invariant distributions. To address this formally, we introduce time into our considerations, allowing each data point to follow a potentially distinct distribution $X_{i}\sim D_{t_{i}}$ linked to the observation time *t*_*i*_. Given the rarity of observing two samples simultaneously, that is, *t*_*i*_ ≠ *t*_*j*_ for all *i* ≠ *j*, it is common to use $D_{i}$ instead of $D_{t_{i}}$ for simplicity (Gama et al., 2014).

This setup aligns with the classical scenario if all *X*_*i*_ share the same distribution, that is, $D_{i}=D_{j}$ for all *i, j*. *Concept drift* takes place when this assumption is violated, that is, $D_{i}\neq D_{j}$ for some *i, j* (Gama et al., 2014).

As argued by Hinder et al. (2020), this definition of concept drift depends on the chosen sample and not the underlying process. This makes drift a non-statistical problem, as one sample may have concept drift while another does not, even though they were generated by the same process within the same time period, but with different sampling frequencies. To address this issue, Hinder et al. (2020) suggest incorporating the statistical properties of time. This is done by using a model of time, denoted as T, instead of a simple index set. The framework assumes a distribution *P*_*T*_ on T that characterizes the likelihood of observing a data point at time *t*, together with a collection of distributions $D_{t}$ for all t∈T, even though only a finite number of time points are observed in practical terms. The combination of *P*_*T*_ and $D_{t}$ forms a *distribution process* (in the literature, this is also referred to as drift process).

**Definition 1**. Let T=[0,1] and $X={\mathbb{R}}^{d}$. A *(post-hoc) distribution process* $\left(D_{t},P_{T}\right)$ from the *time domain* T to the *data space* X is a probability measure *P*_*T*_ on T together with a Markov kernel $D_{t}$ from T to X, that is, for all t∈T, $D_{t}$ is a probability measure on X and for all measurable A⊂X the map $t\mapsto D_{t}\left(A\right)$ is measurable. We will just write $D_{t}$ instead of $\left(D_{t},P_{T}\right)$ if this does not lead to confusion.^1^

Distribution processes are formal models for *data streams*, which consist of independent observations with the only restriction that simultaneous observations follow the same distribution. This differs from a *time series* or *stochastic process* which are randomly sampled functions from time to data where observations can depend on each other, but each time point has only one definite value. Although both describe data and time interdependencies, and observed data can usually be modeled in both setups, their interpretation and areas of application differ significantly (Hinder et al., 2024). For instance, measuring the temperature of an object over time is a time series, yielding a single value per time. Conversely, a stream of ballots qualifies as a distribution process because the distribution is more interesting than an individual vote.

Two particularly relevant types of distributions can be derived from a distribution process: First, by appending a time-stamp to each sample from its arrival, the data follow what we call the holistic distribution D. Second, by aggregating all samples observed within a specific time window W⊂T, the data conform to the mean distribution $D_{W}$ during *W*. Formally, these distributions are defined as follows:

**Definition 2**. Let $\left(D_{t},P_{T}\right)$ be a distribution process from T to X. We refer to the distribution D on X×T which is uniquely determined^2^ by the property $D\left(A\times W\right)=\underset{W}{\int }D_{t}\left(A\right)\text{d}P_{T}\left(t\right)$ for all A⊂X,W⊂T as the *holistic distribution* of $D_{t}$. Furthermore, we call a *P*_*T*_ non-null set W⊂T a *time window* and denote by $D_{W}\left(A\right)=\underset{W}{\int }D_{t}\left(A\right)\text{d}P_{T}\left(t|W\right)=D\left(A\times W|X\times W\right)$ the *mean distribution* during *W*.

A distribution process provides the benefit of data sampling. In contrast, a sample-based arrangement does not allow the creation of a new sample from old ones. Two techniques exist for generating new data from a distribution process. One method involves obtaining i.i.d. samples from the holistic distribution D. These time-stamped data points (*X, T*) are commonly obtained by first randomly selecting an observation time (*T*~*P*_*T*_) and then drawing *X* from the distribution $D_{t}$ with the assumption that *T* = *t*, that is, $X|\left[T=t\right]\sim D_{t}$. Another frequently employed method is generating i.i.d. samples from $D_{W}$ within a specified time window *W*. Importantly, observations within a time window *W* based on D perfectly replicate the distribution described by $D_{W}$. Both methods are formal procedures for obtaining data over time.

Building on the aforementioned definition, we define drift as a property of a data-generating process, not just a sample drawn from it. To account for the statistical nature, a slight adaptation is necessary. We assert that $D_{t}$ exhibits drift if there is a non-zero probability of obtaining a sample with drift. In other words, a sample *X*_1_, *X*_2_, …  will have indices *i* and *j* where


$$\begin{array}{l} {\mathbb{P}}_{X_{i}}\overset{\text{def.}X_{i}}{=}D_{T_{i}}\neq D_{T_{j}}\overset{\text{def.}X_{j}}{=}{\mathbb{P}}_{X_{j}} \end{array}$$

with a probability that is greater than zero. The number of samples does not impact this, due to measure-theoretical considerations, enabling the examination of only two samples for this definition.

** Definition 3**. Let $\left(D_{t},P_{T}\right)$ be a distribution process. We say that $D_{t}$ has *drift* iff


$$\begin{array}{l} {\mathbb{P}}_{T,S\sim P_{T}}\left[D_{T}\neq D_{S}\right]=P_{T}^{2}\left(\left\{\left(t,s\right)\in T^{\;2}|D_{t}\neq D_{s}\right\}\right)>0. \end{array}$$

Here, $P_{T}^{2}$ denotes the product measure of *P*_*T*_ with itself, that is, the measure on $T^{\;2}=T\times T$ that is uniquely determined by $P_{T}^{2}\left(W_{1}\times W_{2}\right)=P_{T}\left(W_{1}\right)P_{T}\left(W_{2}\right)$.

It may be questioned how far this is distinct from having *s* and *t* in T, where $D_{t}\neq D_{s}$. This formally is due to *P*_*T*_ null sets, that is, it is possible that different distribution only occurs at a single point in time, such that we are unable to observe any samples from the other distribution, making it impossible to detect the drift. Therefore, it is a quirk of the formal model rather than a reflection of the actual process.

As mentioned before, we can also use different choices for T. While T=[0,1] might be the best model for clock-time, T={1,…,n} can be used to model different computational nodes, etc. (Hinder et al., 2023c). In particular, if T is at most countable, then drift is equal to the existing of s,t∈T with different distributions $D_{t}\neq D_{s}$ (Hinder et al., 2020).

There are different yet equivalent formalizations of drift (Hinder et al., 2020). These involve situations where there is a non-equality to a standard distribution ($P_{T}\left[D_{t}\neq P\right]>0$ for all distributions *P* on X), non-equality to the mean distribution ($P_{T}\left[D_{t}\neq D_{T}\right]>0$), and distinct distributions for two separate time windows ($D_{W}\neq D_{W^{\prime }}$ for some $W,W^{\prime }\subset T$). However, a very important way to phrase drift is to express it as the dependence between data *X* and time *T*.

** Theorem 1**. *Let* $\left(D_{t},P_{T}\right)$ *be a distribution process from* T *to* X *and let* (X,T)~D *be distributed according to the holistic distribution. Then,* $D_{t}$ *has drift if and only if T* ⫫ *X are not statistically independent, that is, there exist* W⊂T *and* A⊂X *such that* ℙ[*T*∈*W, X*∈*A*] ≠ ℙ[*T*∈*W*]ℙ[*X*∈*A*].

This concept was pivotal in shaping the development of new methods, for example, it was used to reduce the problem of drift detection to independence *X*⫫*T* testing without the necessity of using two windows (Hinder et al., 2020); it was used to describe the location of drift through temporal homogeneity using conditional independence *X*⫫*T*∣*L*(*X*) where *L* are the homogeneous components (Hinder et al., 2021a, 2022a); explaining drift was reduced to the explanation of models that estimate *X* ↦ *T* (Hinder et al., 2023a); the position of anomalies in critical infrastructure was identified as those features *X*_*i*_ that have a particularly strong correlation with time *T* (Vaquet et al., 2024a,b).

### 2.2 Concept drift in supervised and unsupervised setups

In the previous section, we defined drift in the context of data generation. Typically, drift is classified based on its temporal qualities. An *abrupt drift* refers to a sudden change in distribution at a specific time referred to as *change point*, while changes gradually occurring over an interval signify *gradual drift*. During a changing period in *incremental drift*, samples are drawn from both distributions with varying probabilities. *Recurring drift* refers to the reappearance of past distributions, usually due to seasonality. Some authors use alternative nomenclatures, for example, abrupt drift is sometimes referred to as “concept shift,” and gradual or incremental drift as “concept drift.” However, unless specified, we will refer to all those notions simply as “drift.”

Moreover, drift is further categorized based on the modifications made to data and label space distributions. In a data stream of labeled pairs (*X, Y*) within X×Y, where *Y* represents the label, changes in the conditional distribution $D_{t}\left(Y|X\right)$ are referred to as *real drift*, while changes within the marginal $D_{t}\left(X\right)$ are known as *virtual drift* or occasionally *data drift*.

From a statistical perspective, drift in the marginal distribution of *X* and time *T* and the joint distribution of (*X, Y*) and time *T* can be modeled within a common framework despite different interpretations. Real drift can equivalently be described as the conditional statistical dependence of *Y* and *T*, given *X*, that is, *Y* ⫫ *T* ∣ *X* (Hinder et al., 2023d).

Analogous to general machine learning tasks, drift detection can be considered in the *supervised* settings, that is, those that are concerned with conditional distributions usually with respect to a *label* or *target*, and *unsupervised* tasks, that is, those that are concerned with the joint or marginal distributions. While in supervised settings both real and virtual drift might be present, in unsupervised settings only virtual drift has to be considered.

**Figure 1:**
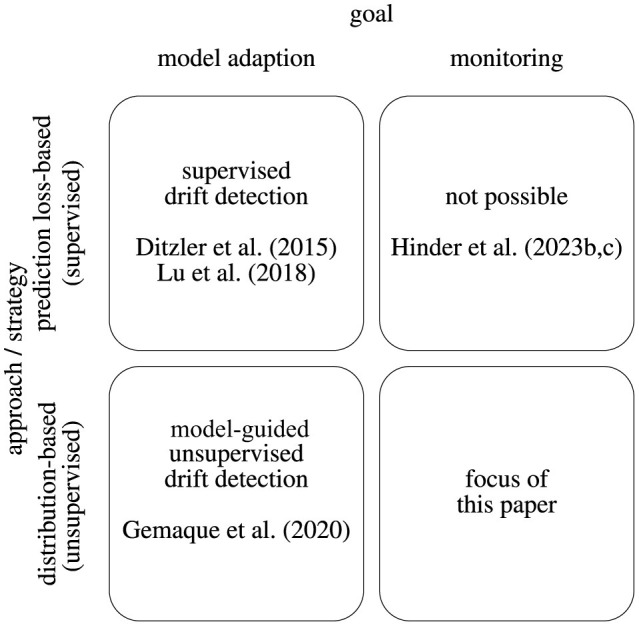
Display of the drift analysis categorization according to the goal and the applied strategy.

Dealing with drifting data streams involves two key objectives: maintaining an accurate learning model despite drift (model adaption) and accurately detecting and characterizing drift in the data distribution (monitoring). In supervised settings, the emphasis is on analyzing model losses and assessing the model's ability to perform prediction tasks (prediction loss-based). In unsupervised settings, more attention is given to the data distribution or data reconstruction (distribution-based). These goals align with two overarching approaches of model adaption and monitoring, resulting in the categorization illustrated in Figure 1.

In supervised environments, model adaptation is typically attained through loss-based tactics, in which updates are guided by the model's capacity to execute tasks. By considering reconstruction losses, such detection strategies can also be used for the unsupervised setup. Many studies examine this supervised strategy (Ditzler et al., 2015; Losing et al., 2018; Lu et al., 2018). However, the connection between model loss, model adaption, and actual drift is rather vague and heavily reliant on the selected model class, the specific properties of the drift, and the setup (Hinder et al., 2023c,d). Therefore, employing loss-based approaches for drift detection in monitoring setups is typically unsuitable.

Unsupervised distribution-based techniques are available for both model fitting and monitoring. We focus on those unsupervised drift detection methods for monitoring tasks, which we discuss further in the following sections. Notably, there is currently no comprehensive survey of drift analysis specifically tailored to the monitoring task, although surveys such as the one by Gemaque et al. (2020) have covered unsupervised drift detection for model adaptation. In addition, Aminikhanghahi and Cook (2017) explore unsupervised change point detection, which is a related problem within the domain of time-series data but is beyond the scope of this discussion.

## 3 Drift detection—setup and challenges

As discussed before, the first important question when monitoring a data stream is *whether (and when)* a drift occurs. The task of determining whether or not there is drift during a time period is called *drift detection*. A method designed to perform that task is referred to as *drift detector*. Surprisingly, most surveys do not provide a formal mathematical definition of drift detectors, so we provide a formalization, first.

One can consider drift detectors as a kind of statistical analysis tool that aims to differentiate between the null hypothesis “for all time points *t* and *s* we have $D_{t}=D_{s}$” and the alternative “we may find time points *t* and *s* with $D_{t}\neq D_{s}$.” More formally, a drift detector is a map or algorithm that, when provided with a data sample *S* drawn from the stream, tells us whether or not there is drift.

We can formalize that such a drift detection model is accurate or valid, respectively, in the following way: (a) the algorithm will always make the right decision if we just provide enough data, or (b) we can control the chance of false positives independent of the stream. This leads to the following definitions:

** Definition 4**. A *drift detector* is a decision algorithm on data-time-pairs of any sample size *n*, that is, a (sequence of) measurable maps $A_{n}:{\left(T\times X\right)}^{n}\to \left\{0,1\right\}$.

A drift detector *A* is *surely drift-detecting* if it raises correct alarms in the asymptotic setting, that is, for every distribution process $D_{t}$ and every δ>0 there exists a number *N* such that for all *n*>*N* we have


$$\begin{array}{l} {\mathbb{P}}_{S\sim D^{n}}\left[A_{n}\left(S\right)=1\left[D_{t}\text{has drift}\right]\right]>1-\delta . \end{array}$$

Notice that the definition is not uniform across multiple streams (or drifts if the method is local in time), that is, for some streams it suffices to have 100 samples to correctly identify drift, for others 10,000 are not enough because the effect is too small. This is not a shortcoming of drift detection but a common scheme for all statistical tests. To cope with that problem we have to take the two kinds of errors into account: A type I error occurs if there is no drift but we detect one (false alarm), and a type II error occurs if there is drift but we do not detect it. As discussed above, avoiding type II errors is not feasible. In addition, as the effect of very mild drifts is usually less severe, missing one might as well be less problematic in practice. Thus, we focus on controlling the type I error.

Controlling the number of false alarms can be stated as follows: Once we provide a certain number of samples, the chance of a false alarm falls below a certain threshold. That number of samples must not depend on the data stream we consider. As this is also fulfilled for the trivial drift detector that never raises any alarms and thus never detects drift, we require that the chance of detecting drift in case there actually is some to be larger than this threshold provided enough data from the stream is available. Here, the amount of required data is stream-specific as discussed above. If a drift detector fulfills these properties at least for some streams, we say that it is valid. If this holds for all streams, then we call the drift detector universally valid. Formally:

** Definition 5**. A drift detector *A* is *valid* on a family of distribution processes 𝔇, if it correctly identifies drift in the majority of cases:


$$\begin{array}{l} \underset{n\to \infty }{\mathrm{limsup}}\underset{D_{t}\in 𝔇,D_{t}\text{ has no drift}}{\sup}{\mathbb{P}}_{S\sim D^{n}}\left[A_{n}\left(S\right)=1\right]\\ \;<\underset{D_{t}\in 𝔇,D_{t}\text{ has drift}}{\inf}\underset{n\to \infty }{\mathrm{liminf}}{\mathbb{P}}_{S\sim D^{n}}\left[A_{n}\left(S\right)=1\right]. \end{array}$$

We say that *A* is *universally valid* if it is valid for all possible streams, that is, 𝔇 is the set of all distribution processes.

Notice that validity does not imply that *A* makes the right decision even if we make use of larger and larger sample sizes. For a concrete case, it makes no statement about the correctness of the output except that it is more likely to predict drift if there actually is drift. This probability, however, holds across all streams independent of the severity of the drift. Thus, for monitoring, we need a drift detector that is universally valid and surely drift-detecting.

One is frequently additionally interested in the time point of the drift. This problem is usually addressed indirectly: If drift is observed in a certain time window, the algorithm will raise an alarm which is then considered as the time point of drift.

## 4 A general scheme for drift detection

**Figure 2:**
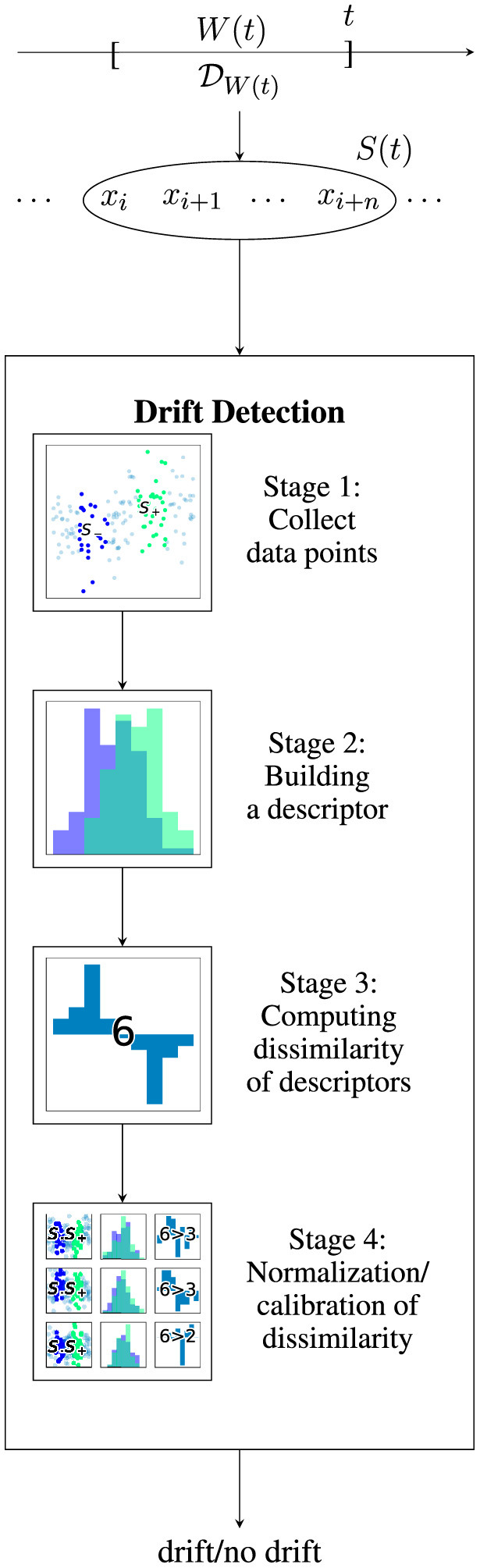
Visualization of drift detection on a data stream: data point *x*_*i*_ was observed at time *t*_*i*_. Given a data stream, for each time window *W*(*t*), a distribution $D_{W\left(t\right)}$ generates a sample *S*(*t*). In this case, *W*(*t*) = [*t*−*l, t*] has a length *l* and thus *S*(*t*) = {*x*_*k*_∣*t*_*k*_∈*W*(*t*)} = {*x*_*i*_, …, *x*_*i*+*n*_}. A drift detection algorithm estimates whether or not *S*(*t*) contains drift by performing a four-stage detection scheme. Illustrated drift detector uses two sliding windows (stage 1), histogram descriptor (stage 2), total variance norm (stage 3), and permutation-based normalization (stage 4).

As discussed before, the goal of drift detection is to investigate whether or not the underlying distribution changes. As visualized in Figure 2, drift detection is usually applied in a streaming setting where a stream of data points is arriving over time. At time *t*, a sample *S*(*t*) containing some data points which are observed during *W*(*t*) and thus are generated by $D_{W\left(t\right)}$ becomes available. On an algorithmic level, existing drift detectors can be described according to the four-staged scheme visualized in Figure 2 following the ideas of Lu et al. (2018).

In this section, we discuss some of the most prominent choices for the stages 1-4 of this drift detection scheme.

### 4.1 Stage 1: acquisition of data

input:  data stream

output: window(s) of data samples, for example, one reference window and one containing the most recent samples

**Figure 3:**
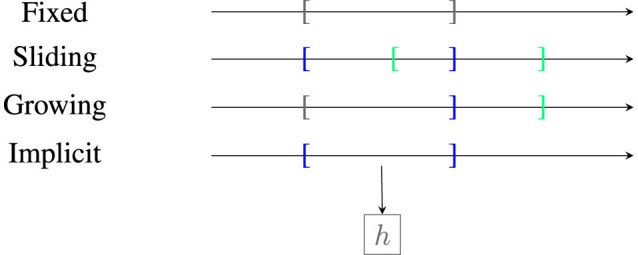
Illustration of reference window types. Area in brackets refers to reference window *W*(*t*), *W*(*s*) for time point *t*<*s*. Border of *W*(*t*) is marked in dark blue, border of *W*(*s*) in light green, and overlapping borders in gray. Here, *h* is a learning model that implicitly stores the data by learning it.

As a first step, a strategy for selecting which data points are used for further analysis needs to be selected. Depending on the strategy used (we will discuss those in Section 5) either one or two windows of the data are selected. Most approaches rely on sliding windows (Lu et al., 2018). As visualized in Figure 3, there are four main categories which differ in how the reference window is updated, for example, fixed until an event, growing, or sliding along the stream or implicit as a summary statistic using a model. We refer to Lu et al. (2018) for a more detailed description. There also exist approaches using preprocessing such as a deep latent space embedding (Vaquet et al., 2021).

### 4.2 Stage 2: building a descriptor

input:  window(s) of data samples

output: possibly smoothed descriptor of window(s)

The goal of the second stage is to provide a possibly smoothed descriptor of the data distribution in the window obtained in stage 1.

Possible descriptors are grid- or tree-based binnings, neighbor-, model-, and kernel-based approaches: Binnings can be considered as one of the simplest strategies. The input space is split into bins, and the number of samples per bin is counted. The bins can be obtained as a grid or by using a decision tree. Decision trees can be constructed randomly, according to a fixed splitting rule (Dasu et al., 2006), or using a criterion that takes temporal structure into account (Hinder et al., 2022b) which can result in better performance.

One can also use a machine learning model's compression capabilities by training the model. This way, the data are stored implicitly (Dwork, 2006; Shalev-Shwartz and Ben-David, 2014; Haim et al., 2022). A query is then used to access the data. Common strategies are discussed in Section 5.

Other versatile, robust, and non-parametric families of methods are offered by a large variety of neighborhood- or kernel-based approaches (Gretton et al., 2006; Harchaoui and Cappé, 2007; Pérez-Cruz, 2009; Liu et al., 2017). In those cases, the information is encoded via (dis-)similarity matrices like the adjacency matrix or kernel matrix.

### 4.3 Stage 3: computing dissimilarity

input:  descriptor of window(s)

output: dissimilarity score

The goal of this stage is to compute a dissimilarity score. Here, different descriptors can be used to compute the same score, and vice versa. Popular choices are the total variation norm (Webb et al., 2017), Hellinger distance (Ditzler and Polikar, 2011; Webb et al., 2016), MMD (Gretton et al., 2006; Rabanser et al., 2019), Jensen-Shannon metric (Salem et al., 2012), Kullback-Leibler divergence (Dasu et al., 2006; Hinder and Hammer, 2023), model loss (Liu et al., 2017; Rabanser et al., 2019), neighborhood intersection (Liu et al., 2017), Wasserstein metric (Zhao and Koh, 2020; Hinder et al., 2022b, 2023a), and mean and moment differences (Hinder et al., 2022b, 2023a). Suitable combinations of dissimilarity measures and descriptors are summarized in Table 1 and will be discussed later.

**Table 1:** Overview of unsupervised drift analysis methods from the literature.

**Strategy**	**Type**	**Method**	**Stage 1 (reference window)**	**Stage 2**	**Stage 3**	**Stage 4**	**DD**	**DP**	**DL**	**DE**
Two-Sample	MB	D3 (Gözüaçık et al., 2019)	Sliding Window	Virtual-Classifier	ROC-AUC	–	✓^*a*^	✗	✗	✗
	ST	Window-KS (Dos Reis et al., 2016; Raab et al., 2020)	Sliding/Fixed Window	Feature-wise Empirical CDF	KS-Statistic	KS-Distribution	✓	✗	✗	✗
	ST	MMD (Gretton et al., 2006; Rabanser et al., 2019)	Sliding Window	Kernel Matrix	MMD	Bootstrap / Permutation Test (or Pearson Statistic)	✓	✗	✗	✗
	ST	HDDDM (Ditzler and Polikar, 2011)	Histogram of Growing Window	Feature-wise Histogram	Hellinger Distance	Adaptive Threshold	✓	✗	✗	✗
	ST	PCA-CD (Qahtan et al., 2015)	Fixated Window	KDE and Histograms on PCA-projection	Maximum Symmetrised Kullback-Leibler Divergence	Page-Hinkley Test	✓	✗	✗	✗
	ST	Drift Magnitude (Webb et al., 2016, 2017)	Sliding Window	Gird Histogram	Total Variation / Hellinger Distance	–	✗	✗	✗	✓
	ST	*kdq*-Tree (Dasu et al., 2006)	Sliding Window	*kdq*-Tree Bins	Kullback-Leibler Divergence	Bootstrap Test	✓	✗	✓	✗
	ST	LDD-DIS (Liu et al., 2017)	Growing with Resampling	*k*-neighborhood	neighborhood Ratio (LDD)	Parametric with Permutation-based Estimate	✓	✗	✓	✗
	ST	LSDD (Bu et al., 2016, 2017)	Growing Window	Density Estimator	*L*^2^-Distance of Densities	Parametric with Bootstrap-based Estimate Test	✓	✗	✗	✗
	ST	MB-DL (Hinder et al., 2023a)	Sliding Window	Random Forest	Kullback-Leibler Divergence to Time Independent Model	Permutation Test	✓	✗	✓	✓^*b*^
	MB	Neighbor Density Comparison (Pérez-Cruz, 2009; Hinder et al., 2022b)	Sliding Window	*k*-Neighbohood	Kullback-Leibler Divergence	–	✓	✗	✗	✗
	MB	Random Proj. Bin. (Rabanser et al., 2019; Hinder et al., 2022b)	Sliding Window	Histogram on Random Projection	Total Variation	–	✓	✗	✗	✗
Meta-Statistic	LB	Model+AdWin (Vaquet et al., 2021)	ML Model	–	Model-Loss	AdWin Statistic	(✓)^*c*^	✓	✗	✗
	ST	ShapeDD (Hinder et al., 2021b)	Consecutive Sliding Windows	Kernel Matrix	MMD	Shape Match + MMD Test	(✓)^*d*^	✓	✗	✗
Block-Based	ST	DAWIDD (Hinder et al., 2020)	Sliding Window	Kernel Matrix	HSIC	Permutation Test	✓	✗	✗	✗
	CL	KCpD (Harchaoui and Cappé, 2007; Arlot et al., 2019)	Offline	Kernel Matrix	Kernel-Variance	None / Slope Heuristic	✓	✓	✗	✗
	MB	Moment Tree Binning (Hinder et al., 2022b)	Sliding Window	Moment Tree	Total Variation	–	✓	✗	✓	✓^*b*^
	MB	Drift Segmentation (Hinder et al., 2021a, 2023a)	Sliding Window	Kolmogorov/Moment Tree	–	–	✗	✗	✓	✓^*b*^

Headers stand for Drift Detection (DD), Drift Pinpointing in time (DP), Drift Localization (DL), and Drift Explanation (DE). Stage 1 current window is a sliding window in all cases. Type refers to the type of normalization strategy used: Statistical Test (ST), model Loss-Based (LB), virtual classifier/Model-Based (MB), and CLustering heuristic-based (CL). ^*a*^ Depends on model but low requirements (Hinder et al., 2022b). ^*b*^ Used by Hinder et al. (2023a) as basis. ^*c*^ Depends on model, dataset, and training (Hinder et al., 2023c,d). ^*d*^ For distant abrupt drifts.

### 4.4 Stage 4: normalization

input:  dissimilarity score

output: normalized dissimilarity

Usually, the scores suffer from estimation errors. This is counteracted by a suitable normalization similar to the *p*-value in a statistical test (Gretton et al., 2006; Liu et al., 2017; Rabanser et al., 2019; Hinder et al., 2020; Raab et al., 2020). Other examples of normalized scales are accuracy or the ROC-AUC (Gözüaçık et al., 2019).

### 4.5 Ensemble and hierarchical approaches

Some authors suggest combining multiple drift detectors (Lu et al., 2018). They are usually arranged in an ensemble, for example, by combining multiple *p*-values after stage 4 into a single one, or hierarchical, for example, by combining a computationally inexpensive but imprecise detector with a precise but computationally expensive validation. Although those approaches differ on a technical level, they do not from a theoretical perspective, as the suggested framework is sufficiently general.

## 5 Categories of drift detectors

**Figure 4:**
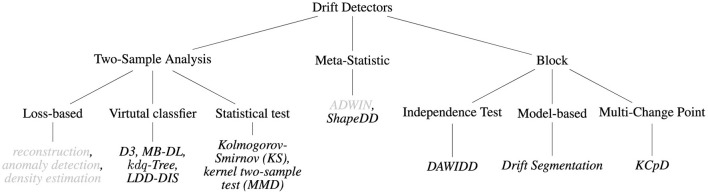
Taxonomy of drift detection approaches discussed in this study. Methods marked in gray are rely on model performance.

So far, we formally defined the properties a drift detection algorithm should fulfill and described on an algorithmic level how different approaches can be implemented. In this section, we focus on concrete approaches. We propose a categorization according to the main strategies of the approaches, relying either on an analysis of two samples, meta-statistics, or a block-based strategy. We present methods organized according to the taxonomy in Figure 4. An overview of the approaches considered in this survey is presented in Table 1.

### 5.1 Two-sample analysis based

The most common type of drift detector exploits that drift is defined as the difference between two time points which can be tested for by statistical two-sample tests. To perform such a test, we split our sample *S*(*t*) into two samples *S*_−_(*t*) and *S*_+_(*t*) and then apply the test to those. The construction of the descriptor, distance measure, and normalization (stages 2–4) are then left to the used testing scheme. In addition to classical statistical tests, there also exist more modern approaches that make use of advanced machine learning techniques.

As stated above, to apply this scheme, we need to split the obtained sample into two sub-samples which are then used for the test. This step is crucial as an unsuited split can have a profound impact on the result. In severe cases, choosing an unsuited split can make the drift vanish and thus undetectable as we consider time averages of the windows. However, there exist theoretical works that suggest that the averaging out does not pose a fundamental problem (Hinder et al., 2021b).

From a more algorithmic perspective, there are essentially three ways the testing procedure is approached. Loss-based and virtual classifier-based approaches rely on machine learning techniques, while statistical test-based approaches rely on statistical tools. We will discuss those in the following.

#### 5.1.1 Loss-based approaches

A large family of loss-based approaches uses machine learning models to evaluate the similarity of newly arriving samples to already received ones. Such models are typically unsupervised or applied without relying on external labels, thus differing from the supervised approach discussed in Section 2.2. However, being reliant on model performance, they face similar pitfalls as prediction loss-based methods (Hinder et al., 2023c). Here, we find it necessary to discuss them due to their widespread popularity. In this case, the reference window (stage 1) is implicitly stored in a machine learning model which is also used as a data descriptor (stage 2). The dissimilarity is usually given by the model loss. It is further analyzed using drift detectors which are commonly used in the supervised setup (Basseville and Nikiforov, 1993; Gama et al., 2004; Baena-Garcıa et al., 2006; Bifet and Gavaldà, 2007; Frias-Blanco et al., 2014) and serve as a normalization (stages 3 and 4).

Several candidates are implementing this strategy. One of the most common model choices are auto-encoders which compress and *reconstruct* the data (Rabanser et al., 2019). Other popular model choices are models like 1-class SVMs or Isolation Forests. Originating from *anomaly detection*, they provide an anomaly score that estimates how anomalous a data point is. Finally, *density estimators*, which are designed to estimate the likelihood of observing a sample, can be applied to detect drift. Here, the idea is that a sample from a new concept is assumed to be unlikely to be observed in the old concept, resulting in a low occurrence probability, high reconstruction error, or anomaly score. Thus, a change in the mean score indicates drift (Yamanishi and Takeuchi, 2002; Kawahara and Sugiyama, 2009).

These methods are quite popular as they are closely connected to supervised drift detection, but they also face similar issues. On a theoretical level, Hinder et al. (2023c,d) showed that for many important models, one can construct streams where the drift is not correctly detected because it is irrelevant to the decision boundary learned by the model class. This claim was further substantiated by empirical evaluations (Hinder et al., 2023c,d; Vaquet et al., 2024a). Thus, such approaches are unsuited for discovery tasks or the monitoring setup. We will therefore only focus very shortly on them.

#### 5.1.2 Virtual-classifier-based

A different approach using machine learning models is based on the idea of virtual classifiers (Kifer et al., 2004; Hido et al., 2008): If a classifier performs better than random guessing, then the class distributions must be different.

**Figure 5:**
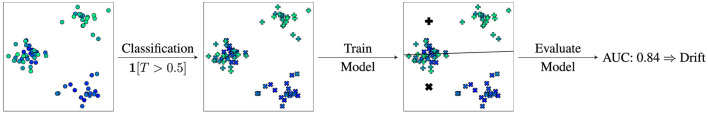
Visualization of virtual classifier-based drift detection. 1. Collect data (moment of arrival is color-coded: dark blue to green), 2. Mark all samples arrived before a certain time as class -1 (cross) and after as class +1 (plus), 3. Train model to distinguish class -1 and +1, and 4. Evaluate model, if performance is better than random chance then there is drift.

This idea can be employed for drift detection as follows (see Figure 5 for an illustration): Store all samples explicitly in two windows (stage 1). Define labels according to reference or current sample, that is, label *x*∈*S*_−_(*t*) as *y* = −1, *x*∈*S*_+_(*t*) as *y* = 1. Use that to train a model (stage 2). The test score then serves as a drift score (stage 3) which is commonly a normalized score (stage 4).

In practice, the usage of *k*-fold evaluation is advised for optimal data usage (Hido et al., 2008; Gözüaçık et al., 2019). Furthermore, statistical learning theory offers guarantees that can be used to derive *p*-values (Kifer et al., 2004; Dries and Rückert, 2009) which, however, are usually rather loose. The used model class is crucial in terms of which drift can be detected and how much data are necessary (Hinder et al., 2022b). It was also shown that for valid split points, many learning models yield surely drift-detecting algorithms and suggested that the resulting algorithms are also universally valid. Furthermore, the chance of choosing an invalid split point is essentially zero (Hinder et al., 2021b). As a candidate of this class, we consider D3 (Gözüaçık et al., 2019).

#### 5.1.3 Statistical-test-based

So far we considered intuitive *ad hoc* approaches. More theory-driven approaches can be derived by considering drift detection as a two-sample testing problem for which formal justification usually exists.

**Figure 6:**
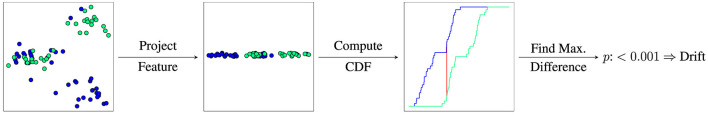
Visualization of Kolmogorov-Smirnov test for drift detection. 1. Collect data (two windows; *S*_−_(*t*) blue and *S*_+_(*t*) green), 2. Feature-wise CDF, 3. Compute largest difference (red line) between CDFs (${\hat{F}}_{t-}$ of *S*_−_(*t*) and ${\hat{F}}_{t+}$ of *S*_−_(*t*)) of feature-wise before and after distribution, and 4. Use analytic *H*_0_ distribution to obtain *p*-value.

Classical statistical tests commonly focus on one-dimensional data. The *Kolmogorov-Smirnov (KS) test* might be the most prominent classical two-sample test (see Figure 6 for an illustration): The test requires two samples (stage 1). It then computes the empirical cumulative distribution function (CDF) (stage 2):


$$\begin{array}{l} {\hat{F}}_{S_{\pm }\left(t\right)}\left(x\right):=\frac{1}{\left|S_{\pm }\left(t\right)\right|}\underset{s\in S_{\pm }\left(t\right)}{\sum }1\left[s\leq x\right] \end{array}$$

The test statistic is given by the maximal distance of the two CDFs (stage 3).


$$\begin{array}{ll} \hat{d}\left(S_{-}\left(t\right),S_{+}\left(t\right)\right) & :=\underset{x}{\sup}|{\hat{F}}_{S_{+}\left(t\right)}\left(x\right)-{\hat{F}}_{S_{-}\left(t\right)}\left(x\right)|. \end{array}$$

Under *H*_0_ the distribution of $\hat{d}$ does not depend on the data distribution (Massey, 1951) and we can compute the *p*-value analytically, serving as a normalized scale (stage 4). All steps can be computed incrementally (Dos Reis et al., 2016).

Applying the test dimension-wise and then taking the minimum extends the method to multiple dimensions (see Algorithm 1). This does not take drift in the correlation into account. It was suggested to use random projection to cope with this problem (Rabanser et al., 2019; Hinder et al., 2022b) which, however, might not work well in practice (Hinder and Hammer, 2023).

**Algorithm 1:**
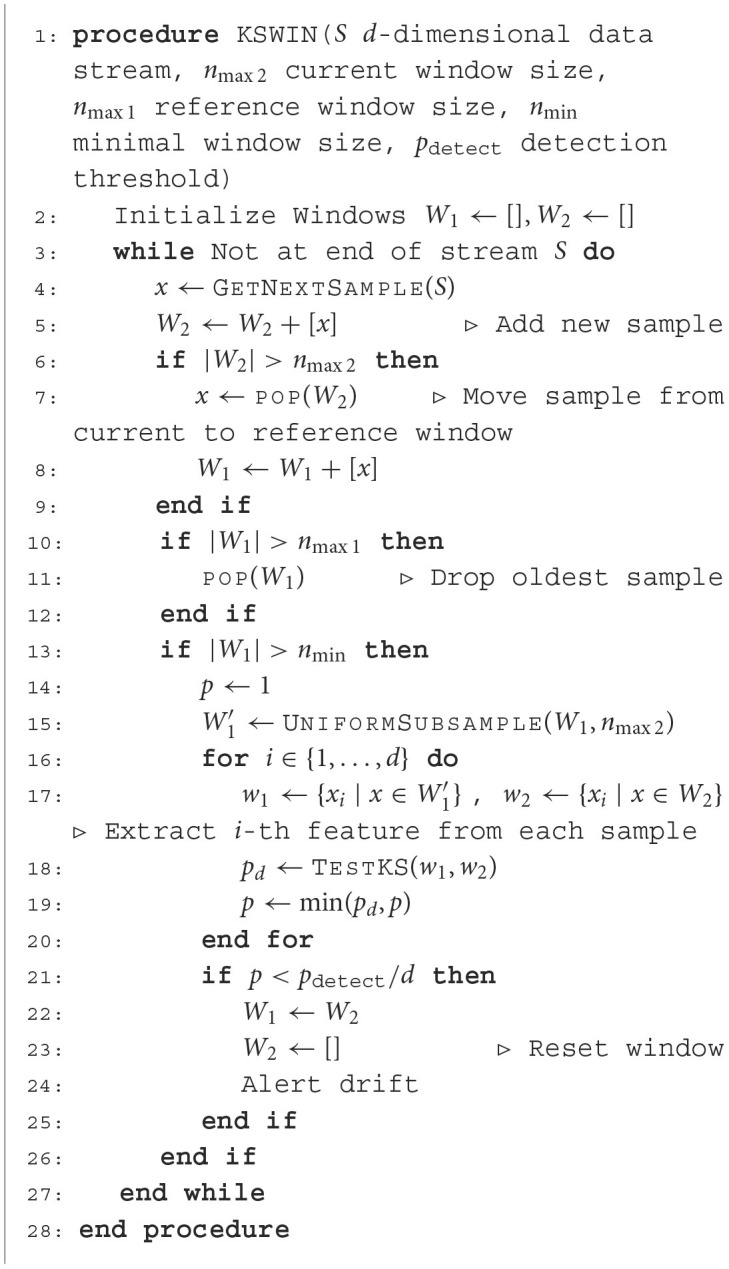
Feature-wise Kolmogorov-Smirnov test on sliding window (Raab et al., 2020).

The *kernel two-sample test* (Gretton et al., 2006; Rabanser et al., 2019) is another important candidate. It is based on the Maximum Mean Discrepancy (MMD) which is similar to virtual classifiers:


$$\begin{array}{l} \text{MMD}\left(P,Q\right):=\underset{∥f{∥}_{H}\leq 1}{\max}|{𝔼}_{X\sim P}\left[f\left(X\right)\right]-{𝔼}_{X\sim Q}\left[f\left(X\right)\right]|. \end{array}$$

In contrast to virtual classifiers, the MMD is computed implicitly using kernel methods. For samples *X*_1_, …, *X*_*m*_~*P, X*_*m*+1_, …, *X*_*m*+*n*_~*Q* and a kernel *k*, we have the estimate ${\hat{\text{MMD}}}_{b}=w^{⊤}Kw$ where *K*_*ij*_ = *k*(*X*_*i*_, *X*_*j*_) is the kernel matrix and $w={\left(\frac{1}{m},\ldots ,\frac{1}{m},-\frac{1}{n},\ldots ,-\frac{1}{n}\right)}^{⊤}$ a weight vector.

Using the kernel two-sample test for drift detection, we again use raw data (stage 1) coming from an arbitrary space. The descriptor is given by the kernel matrix *K* (stage 2) and the score by the MMD (stage 3). For normalization, permutation, or bootstrap testing schemes can be used. Another approach is to use a Pearson curve that is fitted using higher moments. Several more approaches follow similar lines or arguments based on various descriptors or metrics (Rosenbaum, 2005; Harchaoui and Cappé, 2007; Harchaoui et al., 2008, 2009; Chen and Zhang, 2015; Bu et al., 2016, 2017).

As we make use of statistical tests which are valid the drift detector is valid as well (under the same assumptions; see Section 6). Choosing a valid split point is critical but likely from a theoretical point of view (Hinder et al., 2021b, 2022b).

The aforementioned approaches have two main problems: (1) the split point relative to the change point has a huge influence on performance and (2) we face multi-testing problems, that is, the chance of a false positive increase for more tests. Both problems can be addressed by making use of meta-statistics.

### 5.2 Meta-statistic based

So far we have been dealing with two-sample approaches. In a sense, those are the simplest approaches as they consider every time point in the stream separately. This leads to issues such as the multiple testing problem, sub-optimal sensitivity, and high computational complexity. Meta-statistic approaches try to deal with some of these issues by not considering each estimate separately but rather combining the values of several estimates to get better results. To the best of our knowledge, there are only very few algorithms that fall into this category. We will describe two algorithms in detail.

#### 5.2.1 AdWin

**Figure 7:**
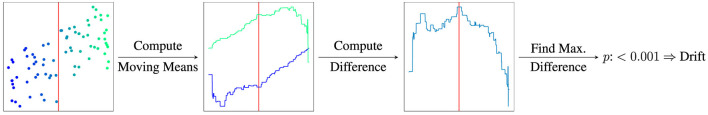
Visualization of AdWin drift detection. 1. Collect data (*S*(*t*) values between 0 and 1; red line marks drift), 2. Compute moving means (μ_*s*−_(*t*) blue μ_*s*+_(*t*) green), 3. Find the largest difference, and 4. Use analytic *H*_0_ distribution to obtain *p*-value.

AdWin (Bifet and Gavaldà, 2007) stands for ADaptive WINdowing and is one of the most popular algorithms in supervised drift detection. It takes individual scores like model losses or *p*-values as input to estimate the actual change point (see Figure 7). The values are stored in a single growing or sliding window *S*(*t*) (stage 1). Then for every time point *s*∈*W*(*t*), the maximal (variance normalized) difference of means is used as a score (stage 2 and 3):


$$\begin{array}{ll} \hat{d}\left(t\right) & =\underset{s\in W\left(t\right)}{\sup}|\mu_{s+}\left(t\right)-\mu_{s-}\left(t\right)|. \end{array}$$

For Bernoulli random variables, corresponding to right and wrongly classified, a *p*-value for the *H*_0_ hypothesis “classification performance only increases” is computed (stage 4). In case of rejection, the moment of drift is the moment of largest discrepancy. Efficient, incremental implementations of this scheme exist. Yet, the connection between model loss and drift is rather vague (Hinder et al., 2023c,d) so it is questionable whether the method is surely drift-detecting or valid.

#### 5.2.2 ShapeDD

The Shape Drift Detector (ShapeDD; Hinder et al., 2021b) is another meta-statistic-based drift detector. In contrast to AdWin, it focuses on the discrepancy of two consecutive time windows, a quantity referred to as drift magnitude (Webb et al., 2017):


$$\begin{array}{l} \sigma_{d,l,D_{\cdot }}\left(t\right)=d\left(D_{\left[t-2l,t-l\right]},D_{\left[t-l,t\right]}\right). \end{array}$$

**Figure 8:**
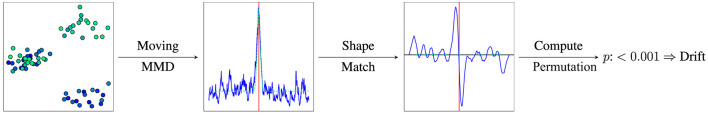
Visualization of ShapeDD. 1. Compute MMD for all time points ($\hat{\sigma }$, dotted line shows theoretically expected shape σ, red line indicated time point of drift), 2. Match obtained shape with theoretically expected one ($\hat{\sigma }*w$), 3. Candidate points are where the match score changes from positive to negative (black line is 0, dots mark candidates), and 4. Compute *p*-values using MMD test at candidate points.

Several choices of distances *d* are allowed making the method widely applicable. Here, we will focus on MMD. The core idea is that in the case of drift, σ not only takes on values larger than 0 but it has a characteristic shape that depends on model parameters only and thus can be detected more robustly (see Figure 8).

Algorithmically, the MMD is computed on two consecutive sliding windows (stages 1–3). Then, the shape function is computed by taking the convolution of $\hat{\sigma }$ with a weight function *w* which is given by *w*(*t*) = −1/*l* for −2*l* ≤ *t* < −*l*, *w*(*t*) = 1/*l* for −*l* ≤ *t* < 0 and *w*(*t*) = 0, otherwise. The points where the shape function changes sign from positive to negative are candidate change points which can then be checked using the usual MMD test (stage 4). All steps can be computed efficiently in an incremental manner. As a consequence of the shape match, most potential split points are not considered in the first place and the candidate points are usually far apart. This reduces the average computational complexity of the method and the chance of encountering false alerts due to multi-testing while also preventing finding the same drift event twice. Furthermore, as the candidate points coincide with the change points up to a known shift, ShapeDD also provides the precise change point (Hinder et al., 2021b) which increases the statistical power of the validation step. This is in contrast to most other two-window approaches. Together with the validity of the kernel two-sample test, this shows that the method is valid and surely drift-detecting for all distribution processes with abrupt drifts that are sufficiently far apart.

However, the characteristic shape is, in fact, an artifact that results from the way the sampling procedure interacts with a single drift event. Thus, it is no longer present if we consider a different windowing scheme (stage 1), several drift events in close succession, or gradual drift. One way to solve the latter issue is to make use of even more advanced meta-statistics that analyze the entire data block at once.

### 5.3 Block-based

In contrast to all other drift detectors considered so far, block-based methods do not assume a split of the data into two windows at any point. Instead, they take an entire data segment into account and analyze it at once.

#### 5.3.1 Independence-test-based

Dynamic Adaptive Window Independence Drift Detection (DAWIDD; Hinder et al., 2020) is derived from the formulation of concept drift as statistical dependence of data *X* and time *T* and thus resolves drift detection as a test for statistical independence. Here, we will make use of the HSIC test (Gretton et al., 2007) which is a kernel method similar to MMD. However, instead of searching for a map that discriminates the two datasets, it searches for a pair of maps that align well, that is, $\sup_{f:T\to R,g:X\to R}\text{cov}\left(f\left(T\right),g\left(X\right)\right)$ where *f* and *g* are found using kernel methods. The test requires a single collection of data points and thus a sliding window (stage 1). If available, the real observation time points can be used; otherwise, it was suggested using the sample id, that is, sample *X*_*i*_ was observed at time *T*_*i*_ = *i*. Using HSIC we compute the kernel matrix of data *K*_*X*_ and time *K*_*T*_ as descriptor (stage 2). The HSIC statistic is then a measure of the dependence of data *X* and time *T* and is estimated by trace(*K*_*X*_*HK*_*T*_*H*), where *H* = *I*−*n*^−1^**11**^⊤^ is the kernel-centering matrix (stage 3). Similar to MMD, the HSIC can be normalized using higher moments which allow fitting a Gamma distribution (Gretton et al., 2007) or a permutation test approach (stage 4). Due to better performance, we make use of the latter. Notice that if the actual observation time is not available, we can use the same time kernel matrix *K*_*T*_ and thus precompute *HK*_*T*_*H* as well as the permutated versions resulting in a drastic reduction in computation time.

DAWIDD makes the fewest assumptions on the data or the drift. This allows for detecting more general drifts but comes at the cost of needing more data—a usual complexity-convergence trade-off. As DAWIDD is again a statistical test, it is also universally valid and surely drift-detecting.

#### 5.3.2 Clustering-based

Clustering offers another block-based approach that structurally falls between independence-test-based and two-sample-test-based approaches. Such methods cluster time points into intervals such that the corresponding data points also form clusters. For the HSIC test, one considers kernalized correlation which can be thought of as fuzzy cluster assignments. In contrast, in clustering, each data point is assigned to a single cluster, which, however, is not predefined by the windows as in the two-sample case. Using a distributional variance measure *V*, such algorithms solve the following optimization problem for a predefined number *n*:


arg  mint0<⋯<tn∑i=0n-1w(ti+1-ti)V(D(ti,ti+1]),

where $T=\left(t_{0},t_{n}\right]$ and *w* is a weighting function.

An instantiation of this approach was proposed by Harchaoui and Cappé (2007) using kernel-variance $V\left(P\right)=\sup_{∥f{∥}_{H}\leq 1}{\text{var}}_{X\sim P}\left(f\left(X\right)\right)$ which can be estimated by $n^{-1}\text{trace}\left(K_{X}H\right)$ (Arlot et al., 2019). The clustering problem can then be solved using dynamic programming. The resulting algorithm is commonly called Kernel Change-point Detection (KCpD). Later on, Arlot et al. (2019) introduced a heuristic to estimate the number of change points *n* using model selection, that is, separating two clusters decreases the objective significantly while splitting one cluster does not.

From a more algorithmic point of view, KCpD searches for blocks along the main diagonal of the kernel matrix so that the mean value of the entries inside the blocks is maximized. The number of blocks is then chosen such that more blocks no longer increase that value significantly. Other algorithms implement similar ideas, for example, Keogh et al. (2001).

Since KCpD is a mainly heuristic method, it is hard to make any statement about its limiting behavior. However, the statistic of the 1-split point case is very similar to the one considered by Hinder et al. (2022b). Furthermore, it is well known that in many cases, kernel estimates have uniform convergence rates. It is thus reasonable that one can derive universally valid surely drift-detecting methods that make use of the same ideas.

#### 5.3.3 Model-based

In addition to the classical kernels which are predefined and not dataset-specific, we can also construct new kernels using machine learning models. In Hinder et al. (2022b), Random Forests with a modified loss function that is designed for conditional density estimation, so-called Moment Trees (Hinder et al., 2021c), are used to construct such kernels. To do so, the model is trained to predict the time of observation *T* from the observation *X*. The resulting kernels show drastic improvements in drift detection tasks (Hinder et al., 2022b). We can also apply this procedure directly to obtain model-based block-based approaches that can be thought of as an extension of the virtual-classifier-based two-window approaches to continuous time by removing the time discretization. The relation between the resulting approaches to DAWIDD is then very similar to the relation of MMD to the model-based two-window approaches like D3.

## 6 Analysis of strategies

So far, we categorized different drift detection schemes and described them according to the four stages discussed in Section 4. In this section, we will consider the different strategies on a more practical level and investigate experimentally in which scenarios which drift detection method is most suitable. For this purpose, we identified four main parameters that describe the data stream and the drift we aim to detect: We investigate the role of the *drift strength*, the influence of drift in *correlating features*, the *data dimensionality*, and the *number of drift events*. To cover the strategies described in Section 5, we select one representative technique per category. As these approaches are structurally similar, from a theoretical viewpoint they carry the same advantages and shortcomings. We will present and discuss our findings in the remainder of this section.

### 6.1 Experimental setup

#### 6.1.1 Datasets

**Figure 9:**
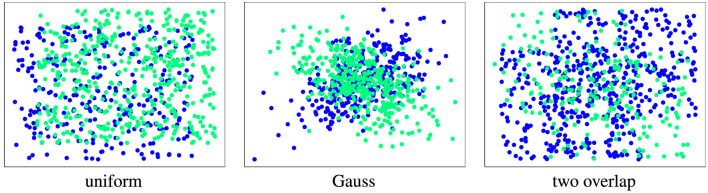
Illustration of used datasets (default parameters, original size). Concepts are color-coded (Before drift: blue, after drift: green).

For our experiments, we consider three 2-dimensional, synthetic datasets with differently structured abrupt drift (see Figure 9). We use modifications of these datasets to evaluate the properties of the discussed drift detection methods.

1. *Uniform*ly sampled from the unit square, drift is introduced by a shift along the diagonal. Intensity is shift length; noise in additional dimensions is uniform.
2. Data sampled from a *Gaussian* (normal) distribution with correlated features, drift flips the sign of the correlation. Intensity is correlation strength; noise in additional dimensions is Gaussian.
3. Data sampled from *two overlapping* uniform squares, drift rotates by 90^*o*^. Intensity is inverse to the size of overlap; noise in additional dimensions is uniform.

Using these base datasets, we generate data streams consisting of 750 samples with drift times randomly picked between *t* = 100 and *t* = 650 by varying the following parameters:

- *Intensity*, default is 0.125.
- *Number* of drift events, default is 1.
- Number of *dimensions* by adding non-drifting/noise dimensions, default is 5, that is, 3 noise dimensions.

#### 6.1.2 Methods^3^

We make use of D3^4^ (used model: Logistic Regression, Random Forest), KS, MMD, ShapeDD, KCpD,^5^ and DAWIDD. For MMD, ShapeDD, KCpD, and DAWIDD, we used the RBF kernel and 2, 500 permutations. This way we cover every major type and sub-type (see Section 5).

For KCpD, we use the extension proposed in Arlot et al. (2019) and choose the smallest α-value to detect a drift as a score. All other methods provide a native score.

The stream is split into chunks/windows of 150 and 250 samples with 100 samples overlapping. Two-sample (split point is midpoint) and block-based approaches are applied to each chunk. Meta-statistic approaches are applied to the stream; then, the chunk-wise minimum of the score is taken.

#### 6.1.3 Evaluation

We run each setup 500 times. The performance is evaluated using the ROC-AUC which measures how well the obtained scores separate the drifting and non-drifting setups. The ROC-AUC is 1 if the largest score without drift is smaller than the smallest score with, it is 0.5 if the alignment is random. Thus, the ROC-AUC provides a scale-invariant upper bound on the performance of every concrete threshold. Furthermore, the ROC-AUC is not affected by class imbalance and thus a particularly good choice as the number of chunks with and without drift is not the same for most setups.

### 6.2 Results

#### 6.2.1 Drift intensity

We evaluate the detectors' capability to detect very small drifts. From a theoretical perspective, we expect that smaller drifts are harder to detect. However, the notion of small here depends on the used detector, for example, the model for D3 or the kernel for MMD, DAWIDD, and KCpD, as well as potential preprocessing (Rabanser et al., 2019; Vaquet et al., 2021; Hinder et al., 2022b; Hinder and Hammer, 2023).

**Figure 10:**
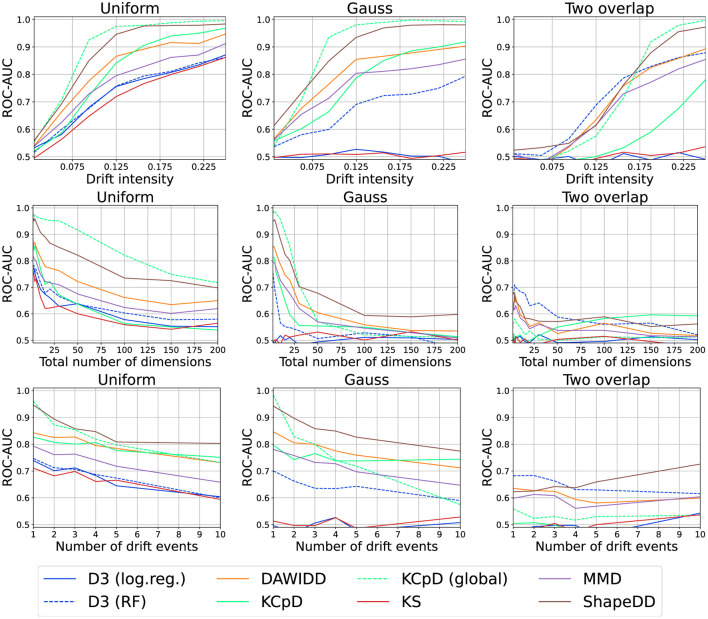
Drift detection performance for various intensities, number of dimensions, and number of drift events.

Our results are visualized in Figure 10. As expected, all methods improve their detection capabilities with increasing drift strengths. ShapeDD performs particularly well. Since it makes use of MMD to test for drift, this implies that the meta-heuristic is quite important. Also for D3, we observe the predicted interplay of model and dataset: For simple datasets, Logistic Regression performs better, and for more complex datasets we need a more advanced model. We will discuss both points later on in more detail. DAWIDD and KCpD also perform quite well, but KCpD requires larger intensities. The global variant of KCpD outperforms all online algorithms closely matched by ShapeDD.

Thus, we suggest incorporating as much domain knowledge into the choice or construction of the descriptor as possible. Furthermore, we recommend the usage of meta-statistic or block-based methods.

#### 6.2.2 Drift in correlating features

Drift can affect the correlation or dependency of several features only, in which case it cannot be detected in the marginal features. We captured this phenomenon in the Gauss and two-overlap datasets. In these cases, KS shows a performance close to random chance and D3 with Random Forests (an axis-aligned model) shows similar issues that cannot be observed for the kernel-based methods.

We thus advise only using methods that make heavy use of feature-wise analysis if drift in the correlations only is either less relevant or very unlikely. If this is not an option, ensemble-based drift detectors that combine feature-wise and non-feature-wise approaches may provide an appropriate solution.

#### 6.2.3 Number of drift events

The number of drift events per time is another relevant aspect in practice. Usually, this number per window is assumed to be comparably small which need not be true in practice. Figure 10 shows the results for different numbers of change points, alternating between two distributions. All drift detectors suffer in this case, which is particularly interesting for global KCpD.

We thus advise making use of block-based drift detectors if several drift events are to be expected. In particular, we suggest not to make use of meta-statistic-based methods unless they can explicitly deal with the setup.

#### 6.2.4 High dimensional data streams

In practice, data are frequently high dimensional with drift only affecting a few features which may cause issues. In Figure 10, we present the results for runs on different numbers of dimensions. Observe that all methods suffer heavily from high dimensionality. For the kernel-based methods, this can be explained by the choice of the RBF kernel, also explaining why global KCpD performs quite poorly. In the case of D3 with Random Forest, this result is somewhat surprising due to the inherent feature selection of tree-based methods. Yet, on Gauss where trees have a harder time exploiting the structure, the method suffers the most.

**Figure 11:**
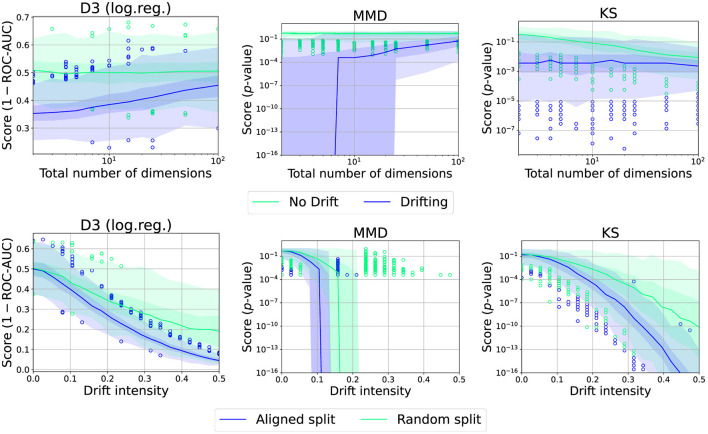
Effect of total number of dimensions or choice of split point for various drift intensities and drift detectors. Graphic shows median (line), 25%−75%-quantile (inner area), min − max-quantile (outer area), and outliers (circles).

We further analyzed the behavior in the case of the uniform dataset with a single drift in the middle and 250 samples (see Figure 11). As can be seen, for D3 and MMD, the drift becomes harder to detect while KS suffers from the multi-testing problem, that is, drift-like behavior emerging by random chance.

Thus, we advise choosing appropriate preprocessing techniques or descriptors to select or construct suited features. Furthermore, in case of high dimensionality with high cost in case of false alarms, one should refrain from using drift detectors that operate feature-wise.

#### 6.2.5 Effect of split point

Meta-statistics and two-window-based methods differ in that the former optimizes the used split point. We study this effect using the uniform dataset with 250 samples, either with optimal or with random split points (see Figure 11). We observe a significant increase in performance which is also more reliable in case of a correct split point. This fits the considerations in Hinder et al. (2021b). We thus advise the user to investigate options to preselect a good candidate split point, either through prior knowledge or by choosing an appropriate algorithm.

#### 6.2.6 D3 model choices

**Figure 12:**
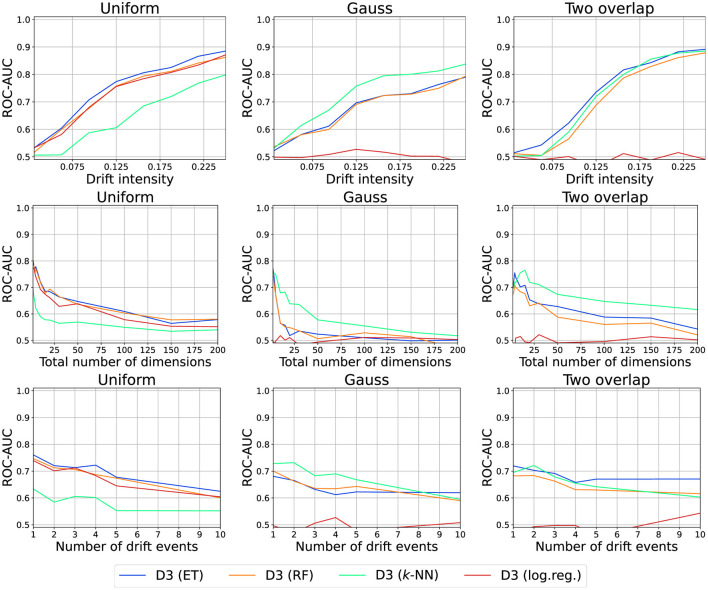
Drift detection performance for various models used by D3.

For D3, the metric is implicitly given by the model making it interesting to study. We consider D3 with different models: Logistic Regression (log.reg.), Random Forests (RF), Extra Tree Forests (ET), and *k*-Nearest Neighbor classifier (*k*-NN; see Figure 12).

The performance is strongly impacted by the model and its interplay with the dataset, for example, *k*-NN is best on Gauss but worst on uniform. Yet, similar models behave alike, for example, ET and RF. Interestingly, feature selection cannot be observed or is ineffective.

Thus, models pose a way to integrate prior knowledge into the detection. This result matches the observations of Hinder et al. (2022b) where the authors argued that the descriptor (stage 2) is more important than the metric (stage 3) derived from it.

#### 6.2.7 Loss-based approaches

**Figure 13:**
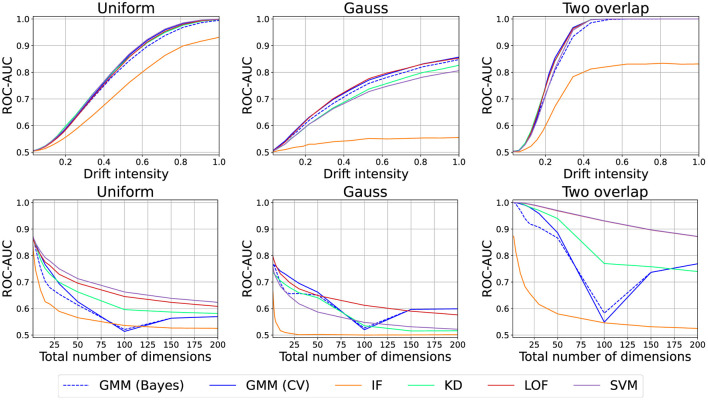
Drift detection performance of various model/loss-based approaches. Experiments use different scale for intensity than previous experiments.

Finally, we also considered outlier- and density/loss-based approaches (Pedregosa et al., 2011): one-class-SVM (SVM; RBF kernel), Local Outlier Factor (LOF; *k* = 10), Isolation Forests (IF), Kernel-Density Estimate (KD; RBF kernel), and Gaussian Mixture Model [GMM; mixture components ≤ 10 cross-validation (CV) or Dirichlet prior (Bayes)]. We use either the outlier score or the sample probability as the drift score. We use the same datasets as before. Here, we use the first 100 samples for training, and the remainder is used for evaluation (see Figure 13). Due to poor performance, we increased the default intensity to 0.5. Otherwise, the results are similar to the other drift detectors.

We thus found additional empirical evidence for the results of Hinder et al. (2023d) which challenge the suitability of loss-based approaches for drift detection from a formal mathematical perspective and therefore suggest the reader not to make use of loss-based approaches.

## 7 Guidelines and conclusion

In this study, we provided definitions of drift and drift detection and discussed the relevance of unsupervised drift detection in the motoring setting. Furthermore, we categorized state-of-the-art approaches and analyzed them based on a general, four-staged scheme (Lu et al., 2018). Table 1 and Figure 4 provided a condensed summary of the proposed taxonomy and summarize how different methods are implemented according to the common staged scheme as visualized in Figure 2.

In addition, we analyzed the different underlying strategies on simple data sets to showcase the effects of various parameters reducing the effect of other dataset-specific parameters. From these experiments, we can derive the following guidelines for the selection and usage of drift detection schemes in monitoring settings:

- A main finding is that as much domain knowledge as possible should be incorporated when designing drift detection schemes. This concerns selecting appropriate preprocessing techniques, constructing and engineering suitable features, and choosing fitting descriptors in stages 1 and 2 for the process.
- Over all experiments, we found that it is advisable to use meta- or block-based methods.
- Choosing good split points is crucial for obtaining good detection capabilities.
- A feature-wise analysis should only be performed if it is expected that the drift does not inflict itself in correlations. Otherwise, relying on multi-variant techniques seems to be the better solution.
- When working with high dimensional data, one should avoid using dimension-wise methodologies, especially if false alarms are costly in the considered application. It might be beneficial to consider feature selection approaches.
- If multiple drifts are expected, applying block-based detectors is particularly suitable.
- Finally, but maybe most importantly loss-based strategies should be avoided when the target of the drift detection is monitoring for anomalous behavior.

Note that our datasets are comparably simple for the sake of controlling a number of parameters. While one might argue that the generality and universality of our findings are of course limited, we think that these controlled experiments provide a first set of guidelines that are valuable as a starting point for developing reliable monitoring pipelines. In particular, we were able to confirm the theoretical considerations of Hinder et al. (2023d) in our experiments.

This study is the first part of a series of studies in which we also cover topics that are closer related to the analysis of concept drift like drift localization and drift explanation. The full series can be found on ArXive (Hinder et al., 2023b).

## Data availability statement

The datasets presented in this study can be found in online repositories. The names of the repository/repositories and accession number(s) can be found at: https://github.com/FabianHinder/One-or-Two-Things-We-Know-about-Concept-Drift.

## Author contributions

FH: Conceptualization, Formal analysis, Investigation, Methodology, Visualization, Writing – original draft. VV: Conceptualization, Formal analysis, Investigation, Methodology, Visualization, Writing – original draft. BH: Conceptualization, Funding acquisition, Supervision, Writing – review & editing.

## Footnotes

^1^All considerations below also work in a very similar way for more general measure spaces. However, for reasons of clarity, we will stick with this special case. This also holds for the restriction of *P*_*T*_ to be a probability measure which is usually specified by the term “*post-hoc*.”

^2^Both existence and uniqueness of D are assured by the Fubini-Tonelli theorem.

^3^The experimental code can be found at: https://github.com/FabianHinder/One-or-Two-Things-We-Know-about-Concept-Drift.

^4^We use the implementation provided by the authors: https://github.com/ogozuacik/d3-discriminative-drift-detector-concept-drift.

^5^We use the implementation provided by Jones and Harchaoui (2020) and Jones et al. (2021).
